# 750 Paddling into danger - Key lessons for a summertime

**DOI:** 10.1093/jbcr/irac012.303

**Published:** 2022-03-23

**Authors:** Timothy Schrire, Mehul Thakkar, Bartlomiej Bednarz

**Affiliations:** North Bristol NHS Trust, Bristol, England; Glasgow Royal Infirmary, London, England; North Bristol NHS Trust, Bristol, England

## Abstract

**Introduction:**

Paddling pools are a source of entertainment for many children. In cooler climates, parents often attempt to heat up the water temperature by adding freshly boiled water into outdoor pools. Our Regional Burns Centre has seen many of these injuries and we wanted to investigate trends and prevalence.

**Methods:**

Utilising the local Burn Injury Database, we searched for paddling pool related injuries which occurred between 2015-2020. We retrieved age, burn size and depth, need for surgical intervention and outcome, as well as circumstances surrounding the injury.

**Results:**

We identified 26 injuries over six years, of which 11 were in 2020. The age of children ranged between 6 months and 13 years old (median 4 years 8 months). The majority, 22 (84%) of patients, had superficial partial thickness injuries. Only five patients (19%) had burns larger than 2% TBSA and only a single patient required general anaesthetic procedure to clean and dress the wounds. Two patients were admitted (length of stay 2- 5 days). Burns affected a range of locations with the majority being lower limbs (11) and upper limbs (8). Head and neck area was affected in 4 cases followed by flank (3), abdomen (2) and buttocks (2). Injuries occurred between April and August and the majority happened during the Friday – Sunday period 18 (69%). All burns occurred between 11:00 and 18:30, with average time being 14:40.

**Conclusions:**

Paddling pools represent a common potential risk to children. With the recent of COVID-19 pandemic leading to closure of the schools, there has been a significant increase in paddling pool sales, resulting in an increase in these injuries and, without relevant education, they are expected to become more common.

